# No Free Lunch—Characterizing the Performance of 6TiSCH When Using Different Physical Layers

**DOI:** 10.3390/s20174989

**Published:** 2020-09-03

**Authors:** Mina Rady, Quentin Lampin, Dominique Barthel, Thomas Watteyne

**Affiliations:** 1 Orange Labs, 38240 Meylan, France; quentin.lampin@orange.com (Q.L.); dominique.barthel@orange.com (D.B.); 2 The National Institute for Research in Computer Science and Automation (Inria), EVA Team, 75012 Paris, France; thomas.watteyne@inria.fr

**Keywords:** 6TiSCH, performance evaluation, OpenWSN, testbed, technology agility

## Abstract

Low-power wireless applications require different trade-off points between latency, reliability, data rate and power consumption. Given such a set of constraints, which physical layer should I be using? We study this question in the context of 6TiSCH, a state-of-the-art recently standardized protocol stack developed for harsh industrial applications. Specifically, we augment OpenWSN, the reference 6TiSCH open-source implementation, to support one of three physical layers from the IEEE802.15.4g standard: FSK 868 MHz which offers long range, OFDM 868 MHz which offers high data rate, and O-QPSK 2.4 GHz which offers more balanced performance. We run the resulting firmware on the 42-mote OpenTestbed deployed in an office environment, once for each physical layer. Performance results show that, indeed, no physical layer outperforms the other for all metrics. This article argues for combining the physical layers, rather than choosing one, in a generalized 6TiSCH architecture in which technology-agile radio chips (of which there are now many) are driven by a protocol stack which chooses the most appropriate physical layer on a frame-by-frame basis.

## 1. Introduction

Applications for low-power wireless technology are numerous, including smart building [1,2], environmental monitoring [3], precision agriculture [4], automated meter reading [5], indoor localization [6], smart grid [7] and predictive maintenance [8].

Several physical layers (PHYs) exist, each finding a different trade-off between energy consumption, datarate and range. One particularly interesting development in low-power wireless technology is the standardization in 2016 of the IEEE802.15.4g amendment [9], which defines additional PHY layers for the older IEEE802.15.4 standard [10]. IEEE802.15.4g defines PHY specifications initially for smart metering utility networks. IEEE802.15.4g defines three families of modulations (Frequency Shift Keying—FSK, Offset Quadrature Phase-Shift Keying—O-QPSK, Orthogonal Frequency Division Multiplexing—OFDM), data rate ranging from 25 kbps to 800 kbps, and two frequency bands (sub-GHz and 2.4 GHz). These options result in a large set of combinations to address a wide variety of applications, from small home installations [11] to kilometer-scale smart agriculture deployments [12]. In the remainder of this article, we call a “PHY” a radio configuration, consisting of a modulation, a data rate and a frequency band.

A traditional approach to having this array of choices is to characterize each PHY, select the one that best corresponds to the application, and build a network solution using that PHY. Two elements allow for a more dynamic approach—technology-agile radio chips and Time Synchronized Channel Hopping (TSCH).

As the processes used in integrated circuits advance, chip manufacturers add more features into their products for the same die size, maintaining low-power operation and competitive pricing. System-on-Chip (SoC), such as Texas Instruments’ CC2538 [13], which combine a micro-controller and a radio in the same die, are now commonplace. Advanced SoC such as the Texas Instruments’ CC2650 [14] contain a co-processor which, combined with a 2.4 GHz front-end, implements either the Bluetooth Low Energy (BLE) or the IEEE802.15.4 standards. Ehile the CC2650 requires that the co-processor be reprogrammed to switch from one to another, the newer nRF52840 [15] from Nordic Semiconductor allow switching between these standards on a frame-by-frame basis. The AT86RF215 from Atmel arguably goes one step further in that it implements the entire set of modulations, data rates and frequency bands defined in the IEEE802.15.4 standard. Using such technology-agile radio chips opens up many possibilities to dynamically match the PHY used to the application.

While early low-power wireless networks used contention-based approach, TSCH, initially developed for industrial applications, is now supported by many commercial products and open-source implementations [16]. In a TSCH network, time is cut into timeslots and a schedule orchestrates all communication; it indicates to every node what to do in each timeslot: transmit, listen or sleep. TSCH is the base principle of standards such as WirelessHART, ISA100.11a, IEEE802.15.4e, and, more recently, 6TiSCH, a set of IETF standards which combines the industrial performance of TSCH with the ease of use of IPv6 [17]. In today’s TSCH networks, for each cell, the schedule indicates the frequency to communicate on, resulting in “channel hopping”, a technique known to efficiently combat external interference and multi-path fading.

Our long-term vision is that low-power wireless networks will combine the technology-agility of modern radio chips which TSCH-like scheduling approaches so nodes dynamically change their PHY layer on a frame-by-frame basis. That is, if the next hop is far, use a long-range (but slower and more energy-hungry) PHY. If the next hop is close, use a fast PHY so the radio can be switched of as fast as possible.

The goal of this article is to get one step closer to this vision by comparing the performance of a 6TiSCH network for different PHY layers. This is a necessary first step towards dynamically changing the PHY layer, the object of our future research. Specifically, we augment the OpenWSN reference 6TiSCH implementation to support the following three PHYs—O-QPSK 2.4 GHz at 250 kbps, FSK 868 MHz option 1 at 50 kbps, OFDM 868 MHz option 1 Modulation and Coding Scheme (MCS) 3 at 800 kbps. The literature indicates that these three PHYs cover the range of possibilities of IEEE802.15.4g: very high data rate with OFDM 868 MHz, very long range with FSK 868 MHz, O-QPSK 2.4 GHz being a balance between range and data rate [12,18,19]. We then conduct a comprehensive experimental campaign on the OpenTestbed, a 42-node testbed of OpenMote B boards [20], a platform which features the AT86RF215 and CC2538 chips. We compare the performance of the network in terms of network formation time, battery lifetime, end-to-end latency and end-to-end reliability.

The remainder of this article is organized as follows. Section 2 surveys the most relevant related work. Section 3 clearly states the problems and lists the contributions of this article. Section 4 introduces the agile extension to the OpenWSN PHY-layer that enabled this research. Section 5 introduces the testbed used for the experimental campaigns of this paper and the methodology of the experiments including the used metrics and KPIs. Section 6 demonstrates the experiment results and KPI evaluations. Finally, Section 7 provides insights and conclusions based on the results.

## 2. Related Work

This section surveys related work on performance improvement and evaluation of the 6TiSCH protocol stack (Section 2.1), performance evaluation of IEEE802.15.4g family of modulations (Section 2.2), and hybrid radio utilization in the 6TiSCH stack (Section 2.3).

### 2.1. Performance Improvement and Evaluation of 6TiSCH

Yang et al. [21] evaluate the performance of the full 6TiSCH stack on the OpenWSN reference architecture in terms of responsiveness to time critical event triggers. They vary the number of active slots in a frame of length 11 slots and measure how packet end-to-end latency is affected.

Theoleyre et al. [22] evaluate the performance of the 6TiSCH stack with the deployment of traffic isolation mechanisms that allow reservation of dedicated slots for certain applications and reservation of shared slots for alarm events. They report end-to-end latency and Packet Delivery Ratio (PDR) under various schedule management strategies, including using uniformly distributed shared cells instead of contiguous (adjacent) shared cells in the slot-frame.

Teles Hermeto et al. [23] execute a performance evaluation of the 6TiSCH protocol stack in an indoor environment with a focus on the stability of the stack performance. They report the end-to-end reliability and the number of parent changes as the network is converging. They also propose a stable link quality metric and a simplified method for schedule inconsistency management.

Ben Yaala et al. [24] evaluate the performance of the 6TiSCH stack in co-located networks. They consider both cases where the coexisting 6TiSCH networks are either synchronized or un-synchronized.

In all of these related works, the PHY used is IEEE802.15.4 O-QPSK 2.4 GHz. Sum et al. [5] are the exception, as they provide an experimental evaluation of IEEE802.15.4e TSCH Medium Access Control (MAC) based on IEEE802.15.4g FSK 868 MHz radio. The experiments report range and performance testing results in terms of PDR and Packet Error Rate in four situations: Line-of-Sight conditions, Non-line-of-sight conditions, tree topology in corridor setting and line topology across buildings.

### 2.2. IEEE802.15.4g Performance Evaluation

Some related work evaluates the performance of IEEE802.15.4g PHYs (without the 6TiSCH stack).

Kojima et al. [25] examine the impact of interference between MR-FSK mode 2 and MR-OFDM option 4 MCS 3. Authors deploy IEEE802.15.4e MAC with multihop capability on top of each PHY layer and measure the impact of the interference between two networks, each running one PHY. Results are reported in terms of the degradation of throughput of each network in the presence of the other interfering network. Authors demonstrates that since the OFDM modulation scheme uses multiple sub-carriers, it performs better than FSK in the presence of frequency selective interference.

Muñoz et al. [1] run an experimental campaign to compare the performance of IEEE802.15.4 O-QPSK 2.4 GHz at 250 kbps, and IEEE802.15.4g OFDM. They show a higher robustness of OFDM, even though it operates as a higher bit rate (800 kbps). They show that, although a radio draws less current when running O-QPSK 2.4 GHz, using OFDM 868 MHz leads to an overall lower power budget as transmission happens much faster.

The same authors also evaluate the performance of all IEEE802.15.4g PHYs [3]. They conduct a complete range-testing campaigns for the 31 PHYs on the four scenarios they consider the most prevalent in outdoor applications: line of sight, smart agriculture, urban canyon, smart metering. Results of the range-tests are reported in terms of PDR measurements, throughput, and electric charge consumption. They demonstrate the longer range of FSK and O-QPSK in the sub-GHz band compared to OFDM options due to their higher receiver sensitivity.

They provide interesting results as to which radio could be better in certain scenarios. This paper goes one step further to address the end-to-end performance of a full 6TiSCH stack using these PHY layers.

### 2.3. Hybrid Radio Utilization

Brachmann et al. [19] integrate multiple PHY layers within the 6TiSCH stack. The authors perform range-testing measurements on multiple modulations: 2-GFSK (at 1.2, 8, 50, and 250 kbps), 4-GFSK (at 1000 kbps), O-QPSK 2.4 GHz (at 250 kbps). They report the link quality in terms of number of motes reached by each mote in the network, and link symmetry. The authors then choose two modulations in the sub-GHz band for integration in the 6TiSCH stack: 2-GFSK at 1.2 kbps for transmitting Enhanced Beacons (EB), 4-GFSK at 1000 kbps for data traffic. This allows for EB sent by the gateway to reach more nodes, resulting in faster and more accurate network-wide synchronization compared to multi-hop transmissions. The slot templates for the PHY layers are designed in accordance with the IEEE802.15.4 standard, and ported on Contiki-NG implementation of 6TiSCH. The authors report on the performance of the multi-PHY network in terms of channel utilization and the distribution of synchronization accuracy in the network, in comparison with the distribution of hop-count. They demonstrate that a faster bit-rate of 4-GFSK at 1000 kbps for data packets diminishes the probability for collision and leads to less than 0.1% channel utilization despite the increased re-transmission rate. At the same time, the slower bit-rate of 2-GFSK at 1.2 kbps for EB packets leads to higher channel occupancy, at 2% duty cycle (leading to violation of the license-free band regulation in many regions).

This research paves the road for a thorough investigation of multi-PHY integration in the 6TiSCH stack. However, it explicitly assumes an inverse correlation between bit-rate and range. This assumption is challenged by the research results in another practical setting in References [1] and [3], which show that a high bit-rate modulation such as OFDM 1 MCS 3 at 800 kbps can offer competitive performance compared to 2-FSK at 50 kbps in terms of range, reliability, and duty cycle (as explained in Section 2.2). This is a significant observation because if long-range radio is needed for EB synchronization as Reference [19] suggests, higher bit-rates are appreciated for EBs in order to minimize energy consumption and also collision probability since EBs are transmitted on shared cells. Furthermore, it still remains unclear whether there are any side-effects to the choice of one radio or another on the end-to-end performance as a whole so that the costs and benefits of each PHY layer choice can be fully assessed.

## 3. Problem Statement and Contributions

While active research proposed various enhancements in 6TiSCH for achieving wire-like reliability and low power consumption, one common assumption is that it uses the IEEE802.15.4 O-QPSK at 2.4 GHz. While O-QPSK 2.4 GHz is appropriate for many applications, the increasing demands for long range (including for environmental monitoring and automated meter reading) has triggered the standardization of longer range PHYs [3]. A problem statement has been proposed in the Internet Engineering Task Force (IETF) detailing the theoretical challenges or side-effects of augmenting 6TiSCH to include different PHYs [18]. Using a different PHY will cause the network to behave differently, leading to different overall performance. For example, a different energy consumption and link cost changes how links to neighbors are evaluated at the link-layer, hence which multi-hop paths are picked by the routing protocol. A different PHY also impacts the number of re-transmissions, the level of interference and the amount of contention is shared cells [26]. Changing the PHY layer triggers subtle changes in the behavior of the protocol stack, resulting in different performance. We believe measuring this difference is best done through a real-world system level evaluation [27]. We show that there are significant benefits of each of the radio options: FSK 868 MHz, OFDM 868 MHz, and O-QPSK 2.4 GHz, yet they all come at a certain cost.

The contribution of this article is three-fold:

- We augment the OpenWSN reference implementation of 6TiSCH to support the three PHY layers (As an online addition to this paper, these extensions have been merged into the main codebase of OpenWSN at https://github.com/openwsn-berkeley and published under an open-source license.).
- We conduct an experimental performance evaluation campaign of the resulting code on the 42-note OpenTestbed. For each PHY, we measure the network formation time, the end-to-end reliability, the end-to-end latency, the estimated battery lifetime of each node, and the memory footprint of the implementation.
- We highlight the advantages and disadvantages of each PHY layer, specifically:
  - Using FSK 868 MHz yields the fastest network formation, the highest end-to-end reliability, and the lowest end-to-end latency, at the cost of a 10× decreased battery lifetime compared to O-QPSK 2.4 GHz.
  - Using O-QPSK 2.4 GHz yields the longest battery lifetime, at the cost of the slowest network formation, the lowest end-to-end reliability, and the highest end-to-end latency.
  - Using OFDM 868 MHz yields an intermediate network formation time, end-to-end reliability, and end-to-end latency at the cost of a 5× decreased battery lifetime compared to O-QPSK 2.4 GHz. In line with Reference [3], a node using OFDM 868 MHz discovers a number of neighbors close to that of FSK 868 MHz despite having the highest data rate. This is because it is more robust against frequency selective interference than FSK 868 MHz  [25] and less vulnerable to Wi-Fi interference than O-QPSK 2.4 GHz  [1].

This shows there is no PHY among our candidates that is best across all metrics.

## 4. A PHY-Layer Agile Extension of OpenWSN

The IETF “IPv6 over the TSCH mode of IEEE802.15.4e” 6TiSCH working group has standardized how to combine TSCH and IPv6 [28]. The 6TiSCH protocol stack is rooted in IEEE802.15.4 at the PHY, and the TSCH mode of IEEE802.15.4e at the link layer. Multi-hop capability is supported at the routing layer via the IPv6 routing protocol for Low-Power and Lossy Networks (RPL). Furthermore, it uses 6LoWPAN as an adaptation layer to interface between the routing layer and the specific operations of the MAC layer such as neighbor discovery, registration, ranking, and maintenance operations that impact how routes are calculated. The 6TiSCH protocol stack has been ported to all main open-source IoT protocol stacks: OpenWSN, Contiki-NG, RIOT and TinyOS [16].

Since it is the reference implementation, and since it is ported to the OpenMote B, we use the OpenWSN implementation in this article. Prior to this work, OpenWSN only supported the IEEE802.15.4 O-QPSK 2.4 GHz PHY. Initial range measurements were conducted using the OpenMote’s AT86RF215 radio [1,3], but the full 6TiSCH stack had never been ported. We therefore extend OpenWSN to support the following PHYs—FSK 868 MHz, OFDM 868 MHz, and O-QPSK 2.4 GHz. This extension consists of three steps—writing the low-level drivers to configure the radio chips appropriately, tuning the durations within the timeslots to each PHY, and providing a clean software abstraction to allow the TSCH implementation to switch between them.

Table 1 lists the main characteristics of the PHY layers tested; TX power and sensitivity number are taken for the chip used.

**Figure 1:**
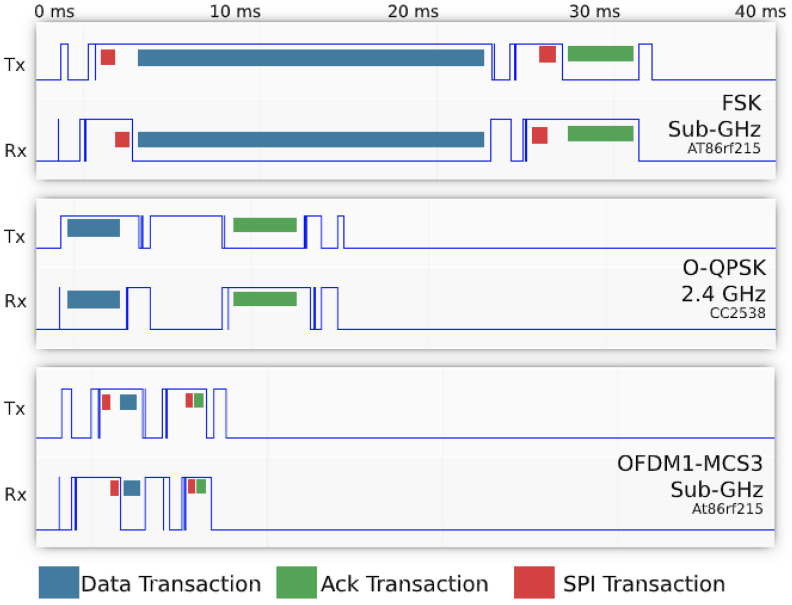
Timeslot templates for the three PHYs. We use a 40 ms timeslot duration in all cases.

We extend OpenWSN with a generic openradio interface. This interface driver implements the same set of functions called by the TSCH state machine, and maps them to that of the radio driver of the PHY being used. We further introduce a timeslot template for each PHY. The “timeslot template” consists of the appropriate timing of the sequence of phases within a timeslot, which we fine tuned using a logic analyzer. The duration of Serial Peripheral Interface (SPI) transactions, the datarate of the communication or the turn-around time of the radio all influence the timeslot template. Figure 1 illustrates the different timeslot templates when transmitting a 100 B data payload with acknowledgement. Red markers highlight the approximate time for SPI communications from the micro-controller to the AT86rf215 radio (none for the CC2538 on-chip radio). A 40 ms slot template duration is used for options, allowing for fair comparison between all three PHYs.

We use a slotframe length of 41 timeslots. Table 2 summarizes the parameters of the stack.

Before this work, the memory footprint of the entire OpenWSN stack was 42 kB. This increases to 77 kB with the additions listed above, in particular with both the CC2538 and AT86RF215 drivers. This is a very small footprint which comfortably fits in modern SoC which all have 512 kB of flash, or more.

## 5. Experiment Setup and Methodology

We use the OpenTestbed [29] to extract the performance of the OpenWSN stack with the extensions detailed in Section 4. This section demonstrates the architecture of the OpenTestbed experimental setup (Section 5.1) and the methodology for the experiment design and performance evaluation (Section 5.2).

### 5.1. The OpenTestbed

**Figure 2:**
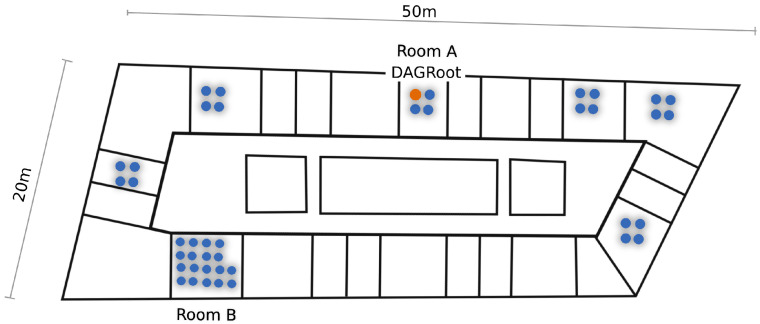
Locations of the 42 motes of the OpenTestbed across an office floor at Inria-Paris.

The OpenTestbed is composed of 42 OpenMote B boards deployed across an office floor at the Inria research center in Paris in groups of 4 (see floorplan on Figure 2). The distribution of the motes happens in clusters of 4–18 motes, mimicking nodes clustered around machines in an industrial setting [8]. The OpenTestbed is built from off-the-shelf components, networked together using the building’s 5 GHz Wi-Fi network (i.e., no dedicated Ethernet network), without requiring back-end servers, and with an open access interface. Interaction with the OpenTestbed is done entirely over Message Queuing Telemetry Transport (MQTT) protocol. The Raspberry Pi single-board computers to which the OpenMote B boards are attached implement a number of commands to reprogram and reset the boards. During an experiment, one can interact (read/write) with the serial port of each OpenMote B board, in real-time, using MQTT messages.

**Figure 3:**
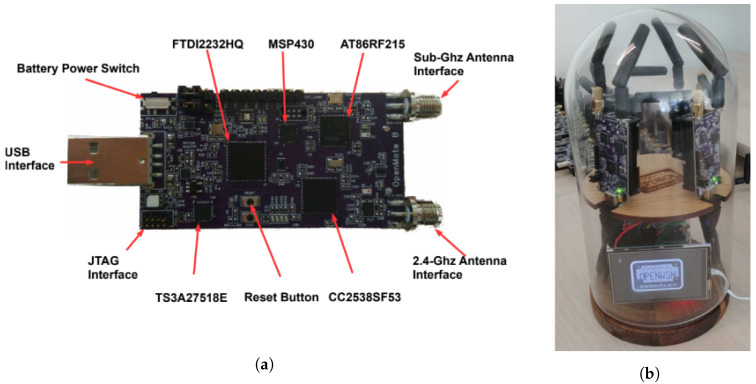
Components of the OpenTestbed experiment setup. (**a**) The OpenMote B, (**b**) the OpenTestbox, part of the OpenTestbed.

As shown in Figure 3, the OpenMote B is an IoT platform which features both a CC2538 (a micro-controller and O-QPSK 2.4 GHz radio) and an AT86RF215 (an FSK 868 MHz and OFDM 868 MHz radio) [20]. There are two antennas: a 2.4 GHz antenna connected to the CC2538, a sub-GHz antenna connected to the AT86RF215. The ARM Cortex-M3 on the CC2538 features 32 kB of RAM and 512 kB of flash. The OpenWSN firmware is loaded onto that micro-controller; that firmware then interacts with the CC2538’s radio directly using the registers, and with the AT86RF215 over SPI. We use a combination of JTAG in-circuit debugging and a logic analyzer to debug the code.

### 5.2. Methodology

We run the OpenWSN network once for each of the three PHYs. During the experiments, the floor is mostly unoccupied, so we do not expect WiFi interference beyond the regular 100 ms beaconing interval of the 8 WiFi access points on that floor [1]. Each time, we load the firmware onto the testbed, then switch on the OpenVisualizer software, which connects to all motes over their serial port (over MQTT). In the OpenVisualizer, we then select the mote that we want to play the role of Directed Acyclic Graph (DAG) root. We always select the same mote, shown in Figure 2, which is positioned at the center of the floor in room A. We then let the network run for 90 min, recording all the data generated by the motes.

We developed a custom-built application which runs at the application layer of each mote. That application sends a data packet every minute containing the following fields:

- a counter, which we use to detect lost data packets,
- the time at which the data packet is generated (Asynchronous Slot Number ASN),
- the DAG rank of the sender,
- the size of the neighbor table of the sender,
- $T_{on}$ how long the sender’s radio has been on since the previous data packet transmission,
- $T_{TX}$ how long the sender’s radio has been on and transmitting since the previous data packet transmission,
- $T_{total}$ the amount of time since the previous data packet transmission,
- the maximum and minimum number of packets in the packet buffer since the previous packet.

After the experiment is done, we use that information to compute the following Key Performance Indicators (these KPIs are recommended by Reference [30]):

- Network Formation Time: how much time elapses between the moment the root is selected and that root has received at least one packet from *each* mote.
- End-to-End Reliability: what portion of all packet generated in the network are received by the root.
- End-to-End latency: how much time elapses between the generation of the packet at the sender, and reception at the root.
- Radio duty cycle: what portion of the time a node’s radio is on; a good indication for its power consumption.

We display the results in three forms:

- Time series. We show the mean and the inter-quartile range of the KPI, with a 3 min sliding window and a 1 s time resolution.
- Cumulative Distribution Function (CDF). We show the cumulative distribution of all data samples at steady state (starting 30 min into the experiment), using 100 bins.
- Probability Distribution Function (PDF). We show the probability distribution of all data samples at steady state using 100 bins. This allows for a more detailed assessment of the distribution of each data set.

## 6. Experimental Results

This section summarizes our key findings, focusing on network formation time (Section 6.1), end-to-end reliability (Section 6.2), end-to-end latency (Section 6.3), and battery lifetime (Section 6.4).

### 6.1. Network Formation Time

The network formation time is measured between the moment the DAG root is selected, and the moment the DAG root has received a data packet from each mote. The network formation process encompasses the time it takes for a motes to synchronize to the network, the time it takes for it to complete a security handshake and the time it takes for it to acquire a rank. It is the time a user would have to wait for their network to be fully functional.

This is a worst case setup, as we turn on all the motes first, and the gateway last. Per the 6TiSCH standard, traffic generated by the motes during their secure handshake uses shared cells. All motes trying to join approximately at the same time will cause a lot of contention of these shared cells, increasing the network formation time.

**Figure 4:**
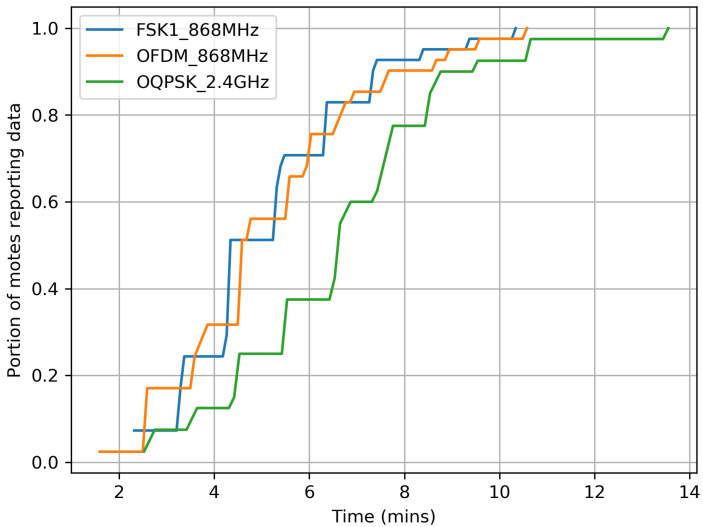
The network tends to form faster when using a longer-range PHY.

Figure 4 shows that the FSK 868 MHz OFDM 868 MHz, and O-QPSK 2.4 GHz network is 90%-formed in 7, 9, and 11 min, respectively. The higher the link budget (i.e., the longer the range), the faster the network forms.

**Figure 5:**
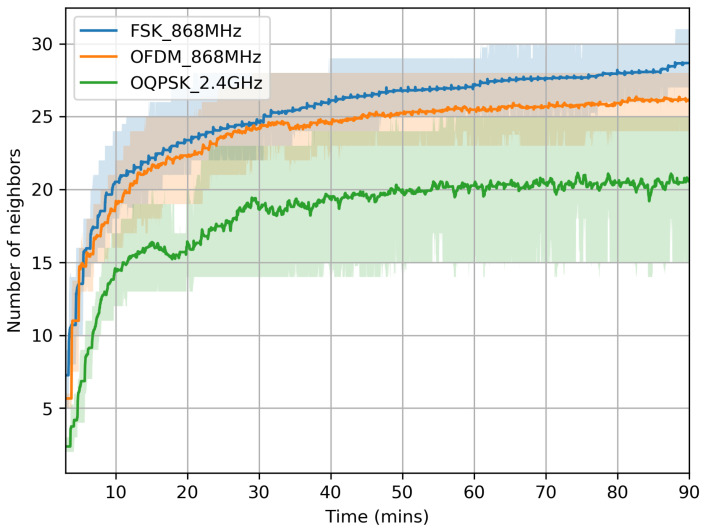
Motes discover more neighbors faster when using a longer-range PHY.

To understand the impact of PHY on network formation time, we plotted in Figure 5 the number of neighbors evolving over time. Because of its long range, a mote using FSK 868 MHz tends to discover more nodes, faster than a node using O-QPSK 2.4 GHz. This observation exactly follows the link budget from Table 1. Discovering many neighbors is helpful in two ways. First, it allows a mote to quickly hear a node that is already part of the network, hence to synchronize quickly. Second, it gives a mote a higher probability of joining through a neighbor closer to the root; this decreases the number of hops necessary for joining.

However, having a longer range increases the risk of neighbor table overflow. For example, if the neighbor table can hold up to 10 entries, what is the appropriate behavior when a mote hears 15 other motes? The challenge is that, without having another mote in its neighbor table, a mote cannot keep statistics to elect the “best” neighbors, and has to to make decisions on partial data, such as a single RSSI value. We note that none of the standards makes clear recommendation for neighbor table grooming.

**Figure 6:**
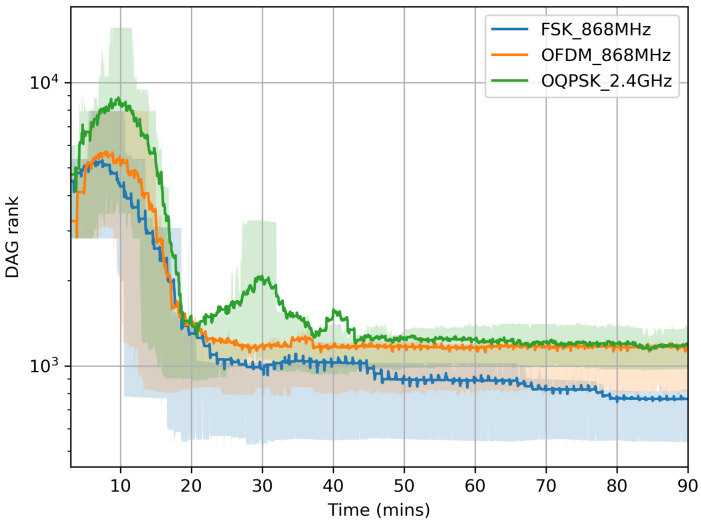
Contention and slow neighbor discovery cause the nodes’ rank to be artificially high at the beginning of network formation.

Figure 6 gives some insights into the network formation itself. It plots how the rank reported by the motes evolves over time. Per the RPL and Minimal Scheduling Function standards [17,31], a mote’s rank is computed using Equation (1), where numTx and numAck are counters of the number of transmission attempts and transmission successes to a neighbor, respectively. minHopIncrease=256 is the rank increase if the link to the mote’s routing parent is ideal. If that link becomes lossy (i.e., numTx/numAck>1), the “cost” associated with the link increases, and the mote’s rank increases accordingly.

(1) $$rank=(\left(3\cdot \frac{numTx}{numAck}\right)-2)-minHopIncrease.$$

The first portion of Figure 6 (t<50 min) shows that the nodes start by having a very high rank. This is caused by two phenomena, combined. First, a mote may not discover its neighbor with the lowest rank immediately, and instead join with a artificially high rank; this resolves over time as the network stabilizes by becoming shallower. Second, all motes attempting to join create contention on the minimal cell, causing transmissions to fail, numTx/numAck to increase, and the motes’ rank to increase. This, again, resolves as the network stabilizes by having less contention on the shared cells. These two phenomena compete—the longer the PHY range, the shallower the initial network, but the higher the contention. Figure 6 shows that, once the network has stabilized, it tends to be shallower (smaller DAG rank) when using a longer-range PHY.

### 6.2. End-to-End Reliability

We call end-to-end reliability the portion of User Datagram Protocol (UDP) datagrams of each mote that reach the root, and use the counter in the datagrams to compute it. Table 3 shows PDR statistics over the last 15 min of the experiments, computed for all motes in the network. We expect close to 100% PDR in all cases (commercial TSCH implementation offer >99.999% PDR [30]).

**Figure 7:**
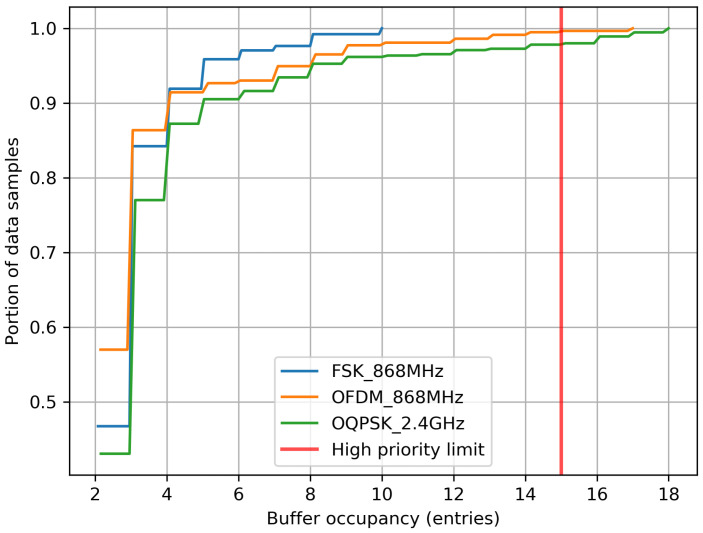
Cumulative Density Function (CDF) of buffer occupancy over the last 15 mins of the experiment. Having more than 15 entries occupied in the buffer (the red line) leads to data packet drops.

Besides possible bugs in our implementation, packet drop might be happening because of queue overflow. As shown in Section 6.1, a lower link budget leads to higher DAG rank, and more relaying, which increases the likelihood of filling up the packet buffer and dropping packets. Figure 7 show the Cumulative Density Function of the queue occupancy values reported by the motes, in the same period corresponding to Table 3. Even though Table 2 correctly indicates the buffer can hold up to 15 packets, 5 additional buffer entries are reserved for control frames such as Enhanced Beacons and acknowledgements. When the number of occupied entries in the buffer exceeds 15, more data packets cannot be admitted into the buffer and are dropped. Motes running O-QPSK 2.4 GHz and OFDM 868 MHz drop packets 3% and 1% of the time, respectively. FSK 868 MHz does not suffer buffer overflow, as the network is much shallower.

### 6.3. End-to-End Latency

We call end-to-end latency the amount of time between the moment a mote generates a new UDP datagram, and the moment it reaches the root. It is computed at the root using a timestamp inside the datagram.

**Figure 8:**
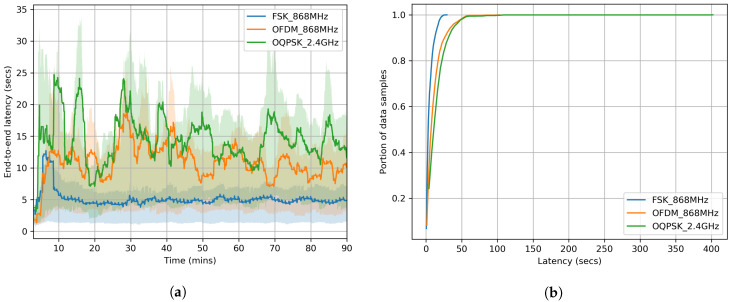

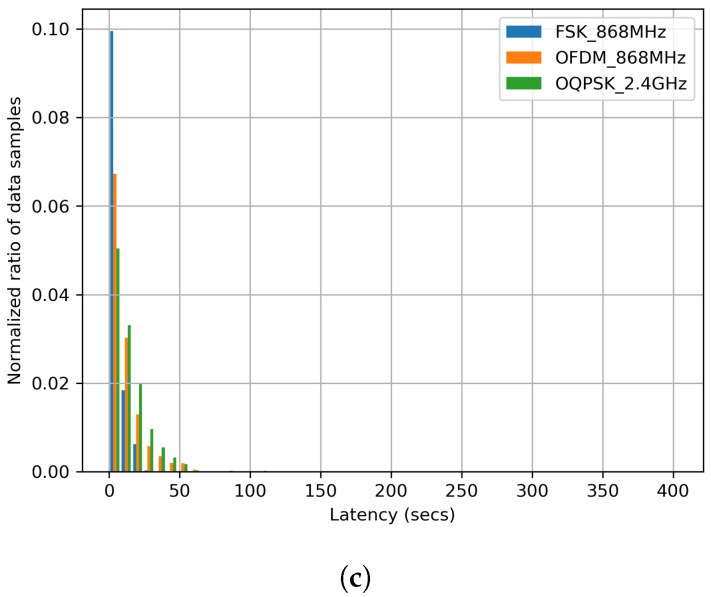
End-to-end latency in the network recorded over the entire 90 min experiments. The longer the PHY range, the lower the latency. (**a**) Time serie; (**b**) Cumulative Density Function (CDF); (**c**) Probability Density Function (PDF).

Figure 8a plots the evolution of latency over time. As shown in Figure 8b, 90% of the data reaches the root after 10, 25, and 35 s for FSK 868 MHz, OFDM 868 MHz and O-QPSK 2.4 GHz, respectively. Figure 8c shows a long-tailed distribution of latency for shorter range PHYs.

With 41 slots per slotframe and 40 ms timeslot, each mote on average gets one transmission opportunity every 1.68 s. Given the increased number of hops for lower range PHYs, the higher latency of O-QPSK 2.4 GHz, compared to that of FSK 868 MHz, was to be expected.

### 6.4. Battery Lifetime

We have the motes report what portion of the time their radio is active (“radio duty cycle”), and what portion of the time the radio is transmitting (“transmit duty cycle”). We then combine that with the current draw of the radio in transmit and receive states, the supply voltage and the energy contained in a battery to compute a battery lifetime. This computation does not take into account the possible current draw of other electronics, and assumes the battery is a perfect bucket of charge (i.e., ideal battery). Even though this is not an accurate prediction of the lifetime of the mote on a real battery, it is good enough to compare the effect on lifetime of the different PHYs (We are aware that current peaks shorten the effective mote lifetime compared to the “ideal battery” lifetime. It turns out that the PHYs leading to the shorter “ideal battery” lifetime in our survey also have the higher peak current. This reinforces our conclusions.).

**Figure 9:**
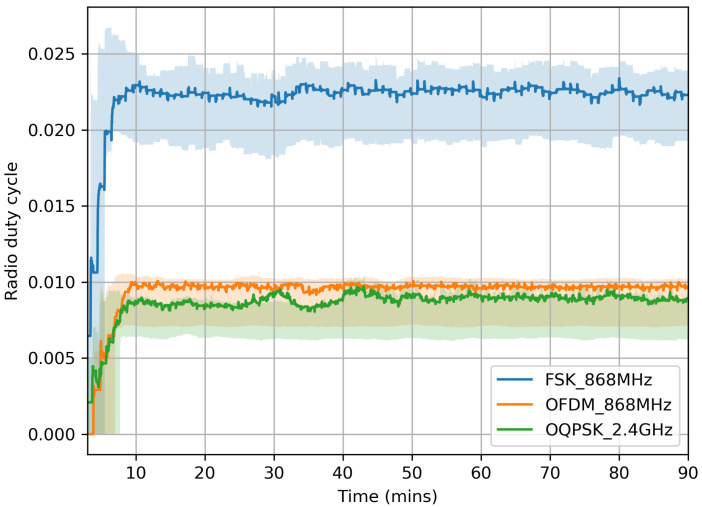
Evolution of the mote’s radio duty cycle over time.

We compute a mote’s radio duty cycle as $T_{on}/T_{total}$ (see Section 5.2). Figure 9 shows the evolution of the duty cycle over time. We expect the duty cycle to decrease with data rate. This does *not* hold for OFDM 868 MHz because of the time to issue SPI commands to the AT86RF215, which can take up to 1 ms as explained in Section 4 and shown in Figure 1.

Given $T_{TX}$ and $T_{on}$, we use Equation (2) to compute reception time $T_{RX}$, the transmit duty cycle $DC_{TX}$ and the receive duty cycle $DC_{RX}$.

(2) $$\left\{\begin{array}{ccc} T_{RX} & = & T_{on}-T_{TX}\;,\\ DC_{TX} & = & \frac{T_{TX}}{T_{total}}\;,\\ DC_{RX} & = & \frac{T_{RX}}{T_{total}}. \end{array}\right.$$

Table 4 details the calculation of battery lifetime, assuming a mote is powered by a pair of AA batteries holding 8.2 Wh of energy. O-QPSK 2.4 GHz exhibits a battery lifetime 5 times larger than OFDM 868 MHz and 10 times larger than FSK 868 MHz. Despite the advantages of FSK 868 MHz and OFDM 868 MHz in range, reliability and latency, their utilization leads to more frequent battery replacement.

## 7. Conclusions

Table 5 summarizes the key performance indicators of our network measured in the experimental campaign. FSK 868 MHz exhibits the best network formation time, end-to-end reliability and end-to-end latency, at the cost of a battery lifetime roughly 10 times lower than O-QPSK 2.4 GHz. OFDM 868 MHz shows balanced results, between FSK 868 MHz and O-QPSK 2.4 GHz. There is no single PHY layer that exhibits the best performance over all KPIs.

*So which PHY should I use?* If choosing a single PHY, which to pick depends on the application. If it is acceptable to change batteries every year or so, FSK 868 MHz appears to be the most appropriate. If battery lifetime is of utmost importance, Table 5 suggests O-QPSK 2.4 GHz is the most appropriate. Finally, for an “in between” performance, choose OFDM 868 MHz.

The problem is that the reasoning in the paragraph above is flawed in at least two ways. First, the results presented in this article, while conducted and presented in a rigorous fashion, only hold for use cases very similar to the deployment shown in Figure 2. There are undoubtedly cases (deeply sparse network, heavily unbalanced deployment, ...) where our results do not hold, and where the “ranking” of the different PHYs is different. Second, even if every precaution is taken to pick the right PHY during the design phase of the network, it is entirely possible that conditions or requirements change during the operation of the network. This could lead to operating with the “wrong” PHY layer, leading to sub-optimal performance.

The real outcome of this paper is not the absolute numbers presented in Table 5. What that table does indicate is that no PHY is the best for all metrics, and that best performance is achieved when *combining* the PHY layers, rather than *picking* one. This result holds when contemplating additional PHY layer, one obvious candidate being LoRa [32]. As alluded to in Section 1, we are at an exciting stage where we have both radio chips which are able to switch PHY on a frame-by-frame basis, and we have the scheduling technology to orchestrate this PHY-layer agility. We argue for technology-agile network, in which each mote keeps track of the quality of its link to its neighbors *for each PHY layer*, and uses the PHY layer most appropriate for each frame. This means a mote may use a different PHY to communicate with different neighbors or to send frames belonging to different classes of traffic. This also means that, in a multi-hop scenario, a packet can travel from source to destination using a different PHY at each hop. In that context, energy efficient neighbor discovery and network consistency are the main challenges, elements we are working on in our current research. 

## Figures and Tables

**Table 1:** The Physical Layer (PHY) layers tested.

	Radio Chip	Data Rate	TX Power	Sensitivity	Link Budget
FSK 868 MHz	AT86RF215	50 kbps	+14.5 dBm	−114 dBm	128.5 dB
OFDM 868 MHz	AT86RF215	800 kbps	+10.0 dBm	−104 dBm	114.0 dB
O-QPSK 2.4 GHz	CC2538	250 kbps	+07.0 dBm	−97 dBm	104.0 dB

**Table 2:** Parameters of the OpenWSN protocol stack.

Parameter	Value
Application traffic period	60 s
RPL DIO period	10 s
RPL DAO period	60 s
neighbor table size	45
packet queue size	15
slotframe length	41
timeslot duration	40 ms
EB probability	10%
Number of radio channels	16
New neighbor RSSI * threshold	−80 dBm
Max num. re-transmissions	15

(*) Received Signal Strength Indicator.

**Table 3:** End-to-end Packet Delivery Ratio (PDR) statistics over all motes in the network, computed over the last 15 min of the experiments.

	Min	Average	Median	Max	StDev
FSK 868 MHz	100.0%	100.0%	100.0%	100.0%	0.0%
OFDM 868 MHz	93.8%	99.8%	100.0%	100.0%	0.9%
O-QPSK 2.4 GHz	73.3%	98.1%	100.0%	100.0%	6.0%

**Table 4:** “Ideal battery” lifetime for each configuration.

	FSK 868 MHz	OFDM 868 MHz	O-QPSK 2.4 GHz
DC	2.100%	0.750%	0.650%
$DC_{TX}$	0.250%	0.038%	0.050%
$DC_{RX}$	1.850%	0.713%	0.600%
$I_{TX}$	62 mA	62 mA	24 mA
$I_{RX}$	28 mA	28 mA	20 mA
Supply voltage	2.5 V	2.5 V	3.0 V
energy per day	0.0141 Wh	0.0033 Wh	0.0015 Wh
battery lifetime	1.6 years	6.9 years	15.1 years

**Table 5:** Summary of the Key Performance Indicator (KPI) measured in our testing. The best values are shown in bold.

	Network Formation	End-to-End Reliability	End-to-End Latency	Battery Lifetime
	**(Section 6.1)**	**(Section 6.2)**	**(Section 6.3)**	**(Section 6.4)**
FSK 868 MHz	**7 min**	**100.00%**	**10 s**	1.6 years
OFDM 868 MHz	9 min	99.84%	25 s	6.9 years
O-QPSK 2.4 GHz	11 min	98.08%	35 s	**15.1 years**
